# Carotid Atherosclerosis, Microalbuminuria, and Estimated 10-Year Atherosclerotic Cardiovascular Disease Risk in Sub-Saharan Africa

**DOI:** 10.1001/jamanetworkopen.2022.7559

**Published:** 2022-04-26

**Authors:** Engelbert A. Nonterah, Daniel Boateng, Nigel J. Crowther, Kerstin Klipstein-Grobusch, Abraham R. Oduro, Godfred Agongo, Shukri F. Mohamed, Palwendé R. Boua, Solomon S. R. Choma, Shane A. Norris, Stephen M. Tollman, Michiel L. Bots, Michèle Ramsay, Diederick Grobbee

**Affiliations:** 1 Navrongo Health Research Centre, Ghana Health Service, Navrongo, Ghana; 2 Julius Global Health, Julius Center for Health Sciences and Primary Care, University Medical Center Utrecht, Utrecht University, Utrecht, the Netherlands; 3 Department of Chemical Pathology, National Laboratory Health Service, Faculty of Health Sciences, University of the Witwatersrand, Johannesburg, South Africa; 4 Department of Epidemiology and Biostatistics, School of Public Health, Faculty of Health Sciences, University of the Witwatersrand, Johannesburg, South Africa; 5 Department of Biochemistry and Forensic Sciences, School of Chemical and Biochemical Sciences, C.K. Tedam University of Technology and Applied Sciences, Navrongo, Ghana; 6 African Population Health Research Centre, Nairobi, Kenya; 7 Institut de Recherché en Sciences de la Santé, Clinical Research Unit of Nanoro, Nanoro, Burkina Faso; 8 DIMAMO Health Demographic Surveillance Site, Department of Pathology and Medical Sciences, School of Health Care Sciences, Faculty of Health Sciences, University of Limpopo, Polokwane, South Africa; 9 MRC/Wits Developmental Pathways for Health Research Unit, Faculty of Health Sciences, University of the Witwatersrand, Johannesburg, South Africa; 10 MRC/Wits Rural Public Health and Health Transitions Research Unit, School of Public Health, Faculty of Health Sciences, University of the Witwatersrand, Johannesburg, South Africa; 11 Sydney Brenner Institute of Molecular Bioscience, Faculty of Health Sciences, University of the Witwatersrand, Johannesburg, South Africa

## Abstract

**Importance:**

Carotid atherosclerosis and microalbuminuria are associated with atherosclerotic cardiovascular disease (ASCVD) but are understudied in sub-Saharan Africa.

**Objective:**

To evaluate the association of carotid atherosclerosis and microalbuminuria with 10-year ASCVD risk in middle-aged sub-Saharan African individuals.

**Design, Setting, and Participants:**

This cross-sectional study conducted analyses of baseline data from the African-Wits-INDEPTH (International Network for the Demographic Evaluation of Populations and Their Health in Low- and Middle-Income Countries) genomic study (AWI-Gen). Women and men aged 40 to 60 years without baseline CVD and drawn from Burkina Faso, Ghana, Kenya, and South Africa were included.

**Main Outcomes and Measures:**

Hypotheses for the analyses were formulated after data collection. The main exposures were carotid atherosclerosis, assessed using carotid intima-media thickness (CIMT) using B-mode ultrasonography, and microalbuminuria, measured using spot urine albumin (SUA) and urine albumin-creatinine ratio (uACR). The main outcome was high ASCVD risk, defined as a 2018 Pooled Cohort Equations score of 7.5% or greater. Associations were estimated using adjusted multivariable logistic regression analyses.

**Findings:**

A total of 9010 participants with a mean (SD) age of 50 (6) years and 4533 (50.3%) women were included. High CIMT, SUA, and uACR were each associated with older age (eg, mean [SD] age of participants with high vs reference range CIMT: 55 [5] years vs 50 [6] years; *P* < .001) and high prevalence of both diabetes and hypertension (eg, hypertension among those with high vs reference range SUA: 213 of 1117 [19.1%] vs 356 of 2549 [14.0%]; *P* < .001). Smokers were likely to have higher vs reference range SUA (210 [18.8%] vs 407 [16.0%]) and uACR (138 of 707 [19.5%] vs 456 of 2797 [16.3%]). Carotid atherosclerosis was common in Burkina Faso (82 of 262 [31.3%]) and Ghana (91 [34.7%]), while microalbuminuria, measured by SUA, was common in Kenya (272 [24.4%]) and South Africa (519 [46.5%]). SUA was associated with higher odds of carotid atherosclerosis (odds ratio [OR], 1.77; 95% CI, 1.04-3.01) compared with uACR (OR, 0.51; 95% CI, 0.27-0.95). Common CIMT, SUA, and uACR were associated with 10-year ASCVD risk, with CIMT having a stronger association with 10-year ASCVD risk in both women (OR, 1.95; 95% CI, 1.78-2.14) and men (OR, 1.73; 95% CI, 1.55-1.93) than SUA (women: OR, 1.29; 95% CI, 1.12-1.43; men: OR, 1.46; 95% CI, 1.26-1.55) and uACR (women: OR, 1.32; 95% CI, 1.10-1.54; men: OR, 1.35; 95% CI, 1.15-1.46).

**Conclusions and Relevance:**

The presence of microalbuminuria measured by SUA may indicate risk of subclinical carotid atherosclerosis and high 10-year ASCVD risk in middle-aged residents of sub-Saharan Africa. These data should be confirmed in longitudinal studies of cardiovascular events.

## Key Points

**Question:**

Are novel cardiovascular disease (CVD) risk factors, such as carotid atherosclerosis (carotid intima-media thickness) and microalbuminuria, associated with with 10-year atherosclerotic CVD (ASCVD) risk in sub-Saharan African adults free of established CVD?

**Findings:**

In this cross-sectional study of 9010 adult participants, microalbuminuria measured using spot urine albuminuria was associated with both carotid atherosclerosis and a high 10-year risk of ASCVD.

**Meaning:**

These findings suggest that spot urine albuminuria can be used in rural and urban sub-Saharan African settings to screen for individuals with a higher risk of ASCVD; however, clinical events data may be needed to confirm these findings.

## Teaser

This cross-sectional study evaluates the association of carotid atherosclerosis and microalbuminuria with 10-year atherosclerotic cardiovascular disease (ASCVD) risk in middle-aged sub-Saharan African individuals.

## Introduction

Cardiovascular disease (CVD) is a leading cause of death and disability worldwide,^1^ with low- and middle-income countries, such as those in sub-Saharan Africa, experiencing 80% of CVD-related global deaths.^2^ Most of these deaths in sub-Saharan Africa occur in younger age groups compared with the rest of the world,^3^ posing health concerns and loss of productive workforce.

Established classical cardiovascular risk factors, such as age, sex, smoking, body mass index, blood pressure, hyperglycemia, and dyslipidemia, are major contributors to CVD in many populations. These factors alone do not explain the total risk for CVD, and several novel factors—such as markers of inflammation, atherosclerosis, and microalbuminuria—contribute to CVD risk.^4,5^ Common carotid intima-media thickness (CIMT) assessed by B-mode ultrasonography is a marker of carotid atherosclerosis that occurs early in life as a low-grade sustained inflammation and progresses with age and the presence of CVD risk factors.^6,7^ Microalbuminuria measured by spot urine albumin (SUA) and albumin-creatinine ratio (uACR) is associated with cardiovascular events, such as congestive heart failure, stroke, and myocardial infarction.^8,9^ The risk is higher in people with hypertension or type 2 diabetes (T2D); therefore in high-income countries, most studies have focused on these populations.

Few studies have examined the associations of microalbuminuria and carotid atherosclerosis with CVD in African populations. This is partly due to the difficulty in assessing CVD risk in Africa as a result of scarce clinical events data. To our knowledge, the PURE (Prospective Urban Rural Epidemiology) and INTERHEART studies are the only studies to have investigated the association of classical risk factors with CVD end points using African populations.^10,11^ These studies were limited by their small sample sizes and, in the case of the PURE study, by selection bias, and neither study examined these novel risk factors. Thus, population-level data on established CVD and associated risk factors is still largely absent in sub-Saharan Africa. This has led to the use of risk equations to assess CVD risk in sub-Saharan African populations. The 2018 revised Pooled Cohort Equation (PCE) is commonly used because of its high accuracy for 10-year atherosclerotic CVD (ASCVD) risk prediction and good performance in African American populations.^12^

To explore cost-effective methods for assessing CVD risk, it is important to examine the association of both classical and novel CVD risk factors, including carotid atherosclerosis and microalbuminuria.^3^ Furthermore, comparing the association of CIMT and microalbuminuria with CVD will offer new insights on factors for population-level CVD screening programs. This study thus measured the association of microalbuminuria with CIMT and the association of both these variables with 10-year ASCVD risk in a large population of middle-aged adults from Burkina Faso, Ghana, Kenya, and South Africa.

## Methods

The Human Hereditary and Health Africa (H3A) Africa-Wits-INDEPTH (International Network for the Demographic Evaluation of Populations and Their Health in Low- and Middle-Income Countries) Partnership for Genomic Studies (AWI-Gen) study received ethics approval from the Human Research Ethics Committee of the University of the Witwatersrand, Johannesburg, South Africa. Ethical approvals were also obtained from relevant ethics bodies in the participating sites. Community engagement, broad consent, and individual informed consent were obtained prior to carrying out study procedures. This report follows the Strengthening the Reporting of Observational Studies in Epidemiology (STROBE) reporting guidelines for observational studies.^13^

### Study Design and Population

We conducted a population-based cross-sectional study using the AWI-Gen cohort between 2013 and 2016.^14,15^ Data were collected from adults residing in health and sociodemographic surveillance sites located in Nanoro (Burkina Faso), Navrongo (Ghana), Nairobi (Kenya), and DIMAMO and Agincourt (South Africa). Additional data were collected from an urban cohort in Soweto, South Africa.^16^

Participants were nonpregnant women and men aged 40 to 60 years with no self-reported previous diagnosis of CVD (based on response to the question “Has a doctor or other health professional ever told you that you had coronary heart disease, angina [also called angina pectoris], heart attack [also called myocardial infarction], or stroke?”). Participants with measured CIMT greater than 1.5 mm were excluded, as this was consistent with plaque and not atherosclerosis.^17^

### Data Collection

A structured questionnaire for obtaining information on sociodemographic variables, clinical history, and behavioral risk factors was administered by trained researchers. Sociodemographic variables included self-reported sex, age at time of data collection, highest level of education attained, and household socioeconomic status using intrasite-generated wealth quintiles. Physical measurements included weight and height for computing body mass index (BMI; calculated as weight in kilograms divided by height in meters squared), waist and hip circumference, and blood pressure. Fasting levels of glucose, lipids, and insulin were measured from blood samples, while albumin and creatinine were measured from urine samples. Data collection tools were standardized across sites with centralized training of researchers. Details of data collection instruments and study procedure have been published elsewhere.^16^

### Estimated 10-Year ASCVD Risk

Estimated 10-year ASCVD risk was computed using the revised 2018 PCE.^12^ This risk equation estimates the 10-year absolute risk of ASCVD, defined as death due to coronary heart disease or nonfatal myocardial infarction or fatal or nonfatal stroke over a 10-year period in people free of established CVD. The PCE permits the derivation of sex- and race-specific estimates of 10-year risk for ASCVD for adults aged 40 to 79 years (which falls within AWI-Gen study’s age category of 40 to 60 years). Variables in the PCE include age, sex, African race, tobacco smoking, total and high-density lipoprotein cholesterol levels, treated or untreated systolic blood pressure, and diabetes status. A PCE score of 7.5% or greater classifies an individual participant at a high ASCVD risk, while less than 7.5% is considered low risk.^18^ Age, sex, and race were self-reported at the time of data collection. We defined smoking as self-reported cigarette and pipe smoking, chewing tobacco use, and snuff use in the 12 months prior to data collection. Blood pressure was measured with the participant seated and with the arm resting on a chair at the level of the heart. An appropriately sized cuff was then applied, and 3 readings were taken with the first discarded, while an average of the last 2 was used as the blood pressure reading. Hypertension was defined as previous diagnosis by a health care professional, taking medication for the condition, or systolic blood pressure of 140 mm Hg or greater and/or diastolic blood pressure of 90 mm Hg or greater. Fasting blood samples were taken for the measurement of fasting glucose and lipid fractions in a central laboratory using standardized procedures. The coefficient of variation of the glucose and lipid measurements ranged between 0.2% and 1.5%. Diabetes status was defined as fasting glucose levels of 126.13 mg/dL or greater (to convert to millimoles per liter, multiply by 0.0555) and/or known diagnosis of diabetes and taking medication at the time of data collection.

### Carotid Atherosclerosis Assessed by CIMT

A LOGIQ e B-mode ultrasound system (GE Healthcare) with a 12L-RS straight transducer was used to measure right and left intima-media thickness of the common carotid arteries (CCAs). The IMT was measured within a 10-mm segment of the CCA starting from 1 cm away from the bulb. Measurements were taken of minimum, maximum, and average distance in millimeters to 2 decimal places. The average of the right and left far wall measurement were averaged to get the outcome of common CIMT. Carotid atherosclerosis was defined as common CIMT of 0.90 mm or greater.^19,20,21,22^

### Microalbuminuria

#### SUA

Midstream urine samples (10-15 mL voided into a sterile collection pot) were collected in accordance with the procedures followed by the World Health Organization.^16^ Samples were centrifuged at 1500 to 2000 × g for 5 minutes and the supernatant aliquoted into cryovials for storage at −80 °C until analysis. Albumin concentrations in urine samples were determined using a turbidimetric assay on a Cobas 501 autoanalyser (Roche Diagnostics). The measuring range was 3 to 400 mg/L for albumin (to convert to grams per deciliter, multiply by 0.001), and the coefficient of variation was less than 2.7%. The use of spot urine as opposed to 24-hour urine collection or albumin-creatinine ratio (ACR) was previously validated in the general population.^23,24^ Microalbuminuria was defined as spot urine albumin concentration of 20 mg/L or greater (or 20-400 mg/L within the confines of the measuring range of the study population).^25^

#### uACR

Urinary albumin concentration was measured with immunoturbidimetry as described previously, and urine creatinine was measured by an IDMS-traceable Jaffe method on the Cobas 501 instrument (Roche Diagnostics). The measuring range for the urine creatinine assay was 3.75 to 550 mmol/L with a coefficient of variation of 2.8%. uACR of more than 3 mg/mmol was defined as microalbuminuria.^25,26^

### Statistical Analysis

Characteristics of the AWI-Gen participants are described using counts and proportions for categorical data and means and SDs for normally distributed continuous data. Baseline characteristics were stratified by carotid atherosclerosis and microalbuminuria. Differences between these groups were determined using Pearson χ^2^ test for categorical data and *t* test for continuous data.

A mixed-effect multivariable logistic regression analysis using country as a random effect was used to determine the association between microalbuminuria and CIMT in the combined sample and for women and men. Adjustments were made for classic CVD risk factors (ie, age, sex [except in the sex-stratified analyses], smoking, BMI, systolic blood pressure, total cholesterol level, high-density lipoprotein cholesterol level, and diabetes), and HIV status.

The associations of carotid atherosclerosis and microalbuminuria with 10-year ASCVD risk were determined using multivariable logistic regression analyses. Owing to differences in their units of measurements, we first generated sex and country specific *z* scores for common CIMT, SUA, and uACR, thus allowing direct comparison of the odds ratios (ORs). We calculated *z* scores using the following equation: (*x* − μ) / σ, where *x* is the raw, individual value; *μ* is the population mean; and *σ* is the population SD. We generated a binary form of PCE based on the American Heart Association definition of high 10-year ASCVD risk as PCE score of 7.5% or greater. We applied adjusted logistic regression analyses to determine the associations of CIMT, SUA, and uACR with high 10-year ASCVD risk. Adjustments were made in a sequential manner using BMI, HIV status, and sociodemographic factors (age, sex [except sex-specific analyses], educational level, and household socioeconomic status), which have been demonstrated to be associated with CVD. Akaike and bayesian information criteria (AIC/BIC) were used to compare the models, and the model with the lowest value was considered a better model. This was further confirmed using the Hosmer-Lemeshow (H-L) goodness of fit test, which determines whether the observed ORs match the expected ORs in the population and population subgroups (ie, countries and sex specific subgroups), and a *P* > .05 was considered a good fit.

For the country level comparisons of the strength of association of CIMT, SUA, and uACR *z* scores with high 10-year ASCVD risk scores, we conducted an inverse variance logistic regression meta-analysis. The best fitted model (fully adjusted model, ie, model 3) was used for these country and sex comparative pooled analyses. The adjusted ORs are presented as forest plots with the within-group variance measured using *I*^2^, where a high *I*^2^ and *P* < .05 indicate high within-group variance. Heterogeneity was measured to assess differences between study groups, with a *P* > .05 indicating low heterogeneity between groups. Associations are reported as ORs with 95% CIs. Statistical significance was set at a 2-tailed *P* < .05. All analyses were conducted using Stata version 14.2 (StataCorp).

## Results

### Study Population Basic Characteristics

The study consisted of 9010 participants with mean (SD) age of 50 (6) years and 4533 (50.3%) women. Presented in Table 1 is a comparison of the characteristics of the study participants categorized by carotid atherosclerosis and microalbuminuria status. A higher proportion of men vs women had carotid atherosclerosis (145 of 262 [55.4%] vs 117 [44.6%]; *P* = .02) and microalbuminuria, assessed by SUA, (581 of 1117 [52.0%] vs 536 [48.0%]; *P* = .01). Participants with tertiary level education, compared with those with no formal education, had lower levels of carotid atherosclerosis (8 [3.1%] vs 146 [55.9%]; *P* < .001) and microalbuminuria assessed by SUA (33 [3.0%] vs 358 [32.1%]; *P* = .03). High CIMT, SUA, and uACR were each associated with older age (eg, mean [SD] age of participants with high CIMT vs reference range CIMT: 55 [5] years vs 50 [6] years; *P* < .001). Smokers were likely to have higher vs reference range SUA (210 [18.8%] vs 407 [16.0%]) and uACR (138 of 707 [19.5%] vs 456 of 2797 [16.3%]). Participants from Ghana and Burkina Faso had higher levels of carotid atherosclerosis (Ghana: 91 [34.7%]; Burkina Faso: 82 [31.3%]) compared with participants from Kenya and South Africa (Kenya: 22 [8.4%]; South Africa: 67 [25.6%]) and the opposite was observed for microalbuminuria (Kenya: 272 [24.4%]; South Africa: 519 [46.5%]; Ghana: 151 [13.5%]; Burkina Faso: 175 [15.7%]). Participants with carotid atherosclerosis, microalbuminuria and high ASCVD risk had higher proportions of hypertension and diabetes (eg, hypertension among those with high vs reference range SUA: 213 [19.1%] vs 356 [14.0%]; *P* < .001).

**Table 1.** Distribution of Sociodemographic and Cardiometabolic Variables by Carotid Atherosclerosis and Microalbuminuria

Variable	Carotid atherosclerosis			SUA			uACR		
	Participants with reference range CIMT (n = 8611), No. (%)	Participants with high CIMT (n = 262), No. (%)^a^	*P* value	Participants with reference range SUA (n = 2549), No. (%)	Participants with high SUA (n = 1117), No. (%)^b^	*P* value	Participants with reference range uACR (n = 2797), No. (%)	Participants with high uACR (n = 707), No. (%)^c^	*P* value
Age, mean (SD), y	50 (6)	55 (5)	<.001	50 (6)	51 (6)	.007	50 (6)	51 (6)	<.001
Sex									
Women	4350 (50.5)	117 (44.6)	.02	1370 (53.8)	536 (48.0)	.01	1442 (51.6)	361 (51.1)	.81
Men	4261 (49.5)	145 (55.4)		1179 (46.3)	581 (52.0)		1355 (48.4)	346 (48.9)	
BMI, mean (SD)	24.1 (6.1)	24.3 (6.2)	.62	24.8 (6.0)	24.8 (6.1)	.95	24.8 (6.4)	25.1 (6.7)	.24
Household SES									
Quartile 1, poorest	1185 (13.8)	32 (12.2)	.47	368 (14.5)	130 (11.6)	.18	394 (14.1)	84 (11.9)	.52
Quartile 2, poorer	1695 (19.7)	53 (20.2)		531 (20.9)	255 (22.8)		590 (21.1)	154 (21.8)	
Quartile 3, poor	1597 (18.4)	40 (15.3)		455 (17.9)	200 (17.9)		482 (17.3)	135 (19.1)	
Quartile 4, less poor	1889 (22.0)	57 (21.8)		541 (21.2)	235 (21.0)		598 (21.4)	150 (21.2)	
Quartile 5, least poor	2259 (26.3)	80 (30.5)		652 (25.6)	297 (26.6)		731 (26.2)	184 (26.0)	
Highest level of education									
No formal education	3335 (38.8)	146 (55.9)	<.001	909 (35.7)	358 (32.1)	.03	995 (35.6)	216 (30.6)	.03
Primary	2493 (29.0)	61 (23.4)		793 (31.2)	387 (34.7)		875 (31.3)	245 (34.7)	
Secondary	2449 (28.5)	46 (17.6)		740 (29.1)	339 (30.4)		811 (29.0)	224 (31.7)	
Tertiary	319 (3.7)	8 (3.1)		104 (4.1)	33 (3.0)		113 (4.0)	22 (3.1)	
Country									
Burkina Faso	1962 (22.8)	82 (31.3)	<.001	583 (22.9)	175 (15.7)	<.001	631 (22.6)	100 (14.1)	<.001
Ghana	1632 (19.0)	91 (34.7)		234 (9.2)	151 (13.5)		270 (9.7)	96 (13.6)	
Kenya	1886 (21.9)	22 (8.4)		678 (26.5)	272 (24.4)		738 (26.4)	165 (23.3)	
South Africa	3131 (36.4)	67 (25.6)		1056 (41.4)	519 (46.5)		1158 (41.4)	346 (48.9)	
Components of PCE									
Smoking	1541 (17.9)	39 (14.9)	.21	407 (16.0)	210 (18.8)	.04	456 (16.3)	138 (19.5)	.04
SBP, mean (SD), mm Hg	123 (22)	133 (25)	<.001	125 (22)	134 (27)	<.001	125 (22)	134 (27)	<.001
Hypertension and taking medication	946 (11.0)	46 (17.6)	.01	356 (14.0)	213 (19.1)	<.001	398 (14.2)	137 (19.4)	.01
Diabetes^d^	531 (6.2)	29 (11.1)	<.001	171 (6.7)	128 (11.5)	<.001	181 (6.5)	99 (13.9)	<.001
Total cholesterol, mean (SD), mg/dL	147.3 (43.2)	145.4 (46.5)	.49	151.6 (43.0)	154.0 (44.8)	.11	151.3 (42.9)	155.5 (45.6)	.03
HDL-C, mean (SD), mg/dL	46.0 (16.1)	43.5 (14.2)	.01	46.4 (16.4)	47.8 (19.4)	.02	46.6 (16.9)	47.3 (18.8)	.32
10-y ASCVD risk ≥7.5%	912 (10.6)	69 (26.3)	<.001	319 (12.5)	262 (23.5)	<.001	345 (12.3)	195 (27.6)	<.001

Abbreviations: ASCVD, atherosclerotic cardiovascular disease; BMI, body mass index (calculated as weight in kilograms divided by height in meters squared); CIMT, common carotid intima-media thickness; HDL-C, high-density lipoprotein cholesterol; PCE, Pooled Cohort Equation; SBP, systolic blood pressure; SES, socioeconomic status in wealth quintile Q1-Q5; SUA, Spot urine albuminuria; uACR, urine albumin-creatinine-ratio.

SI conversion factors: To convert cholesterol and HDL-C to millimoles per liter, multiply by 0.0259.

^a^ High CIMT defined as common CIMT of 0.9 mm or greater.

^b^ High SUA defined as SUA concentration of 20 mg/L or greater.

^c^ High uACR defined as uACR greater than 3 mg/mmol.

^d^ Diabetes was defined as previous diagnosis by a health care professional (which excluded gestational diabetes), taking medication for the condition, or a fasting blood glucose level of 126.13 mg/dL or greater (to convert to millimoles per liter, multiply by 0.0555).

### Association of Microalbuminuria With CIMT

**Figure 1.**
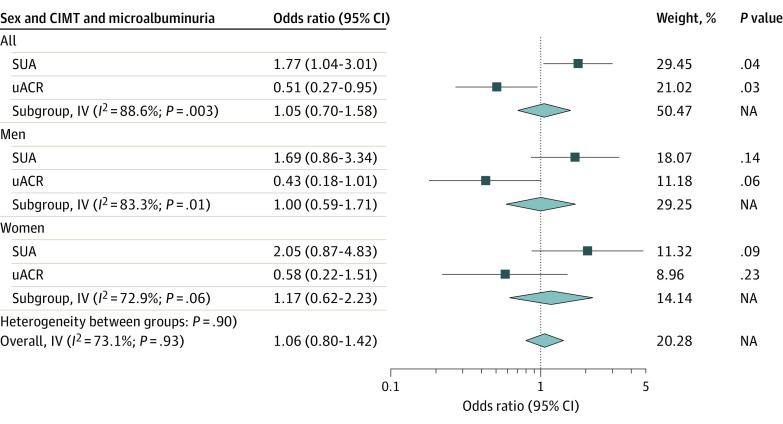
Association of Microalbuminuria With Carotid Intima-Media Thickness (CIMT) in the Combined Sample and in Women and Men IV indicates inverse variance; NA, not applicable.

The associations of microalbuminuria with CIMT in the combined sample and in women and men are presented in Figure 1. In the combined population, high SUA was significantly associated with higher risk of carotid atherosclerosis (adjusted OR, 1.77; 95% CI, 1.04-3.01), but this association was not significant when disaggregated by sex. uACR was not associated with a higher risk for elevated CIMT in women and men or in the combined population. There was significant within-country variance in measured SUA and uACR, but there was no heterogeneity between women and men (eFigure in the Supplement).

### Association of Carotid Atherosclerosis and Microalbuminuria With High 10-Year ASCVD Risk

Presented in Table 2 are the associations of carotid atherosclerosis or microalbuminuria with high 10-year ASCVD risk, stratified by sex. In the combined population, common CIMT and both measures for microalbuminuria were associated with 10-year ASCVD risk in all the models, including the disaggregated models for women and men. Thus, in model 3 of the combined population, participants with higher common CIMT had higher odds of 10-year ASCVD risk (OR, 1.85; 95% CI, 1.75-1.99) compared with SUA (OR, 1.37; 95% CI, 1.24-1.45) and uACR (OR, 1.34; 95% CI, 1.18-1.43). This observed pattern was similar in women and men (Table 2). In the fully adjusted model 3 for combined population, men and women were well fitted due to lower AIC/BIC values and H-L tests with *P *values greater than .05.

**Table 2.** Association of CIMT and Microalbuminuria With High CVD 10-Year Risk in Combined Study Populations and in Women and Men

Models	Common CIMT *z* score			SUA *z* score			uACR *z* score		
	OR (95% CI)	*P* value	AIC/BIC	OR (95% CI)	*P* value	AIC/BIC	OR (95% CI)	*P* value	AIC/BIC
All^a^									
Model 1	1.55 (1.46-1.65)	<.001	5973.58	1.29 (1.20-1.39)	<.001	3163.38	1.27 (1.16-1.39)	<.001	2983.43
Model 2	1.93 (1.80-2.07)	<.001	5264.00	1.32 (1.22-1.42)	<.001	2908.98	1.28 (1.16-1.40)	<.001	2752.09
Model 3	1.85 (1.73-1.99)	<.001	5196.41	1.37 (1.24-1.45)	<.001	2809.06	1.34 (1.18-1.43)	<.001	2653.46
H-L	χ^2^, 7.63	.47	NA	χ^2^, 6.46	.58	NA	χ^2^, 6.83	.56	NA
Women^b^									
Model 1	1.62 (1.50-1.75)	<.001	3596.79	1.24 (1.11-1.38)	<.001	1929.75	1.22 (1.06-1.42)	.01	1797.27
Model 2	2.01 (1.83-2.20)	<.001	3214.92	1.21 (1.08-1.36)	.01	1811.38	1.23 (1.05-1.44)	.01	1689.03
Model 3	1.95 (1.78-2.14)	<.001	3157.65	1.29 (1.12-1.43)	<.001	1750.11	1.32 (1.10-1.54)	.02	1624.27
H-L	χ^2^, 5.72	.68	NA	χ^2^, 11.84	.16	NA	χ^2^, 10.70	.22	NA
Men^b^									
Model 1	1.52 (1.38-1.68)	<.001	2245.76	1.43 (1.30-1.57)	<.001	1153.68	1.34 (1.19-1.52)	<.001	1124.66
Model 2	1.83 (1.64-2.03)	<.001	2058.06	1.40 (1.27-1.55)	<.001	1107.17	1.30 (1.52-1.47)	<.001	1076.01
Model 3	1.73 (1.55-1.93)	<.001	2052.30	1.46 (1.26-1.55)	<.001	1079.40	1.35 (1.15-1.46)	<.001	1049.15
H-L	χ^2^, 13.35	.10	NA	χ^2^, 8.55	.38	NA	χ^2^, 8.66	.37	NA

Abbreviations: AIC, Akaike information criterion; BIC, Bayesian information criterion; CIMT, carotid intima-media thickness; H-L, Hosmer-Lemeshow; NA, not applicable; OR, odds ratio; SUA, spot urine albuminuria; uACR, urine albumin-creatinine-ratio.

^a^ Model 1 was the unadjusted model; model 2, adjusted for sex, education, and country; model 3, adjustments as for model 2 plus household socioeconomic status, body mass index, and HIV status.

^b^ Model 1 was the unadjusted model; model 2, adjusted for education and country; model 3, adjustments as for model 2 plus household socioeconomic status, body mass index, and HIV status.

**Figure 2.**
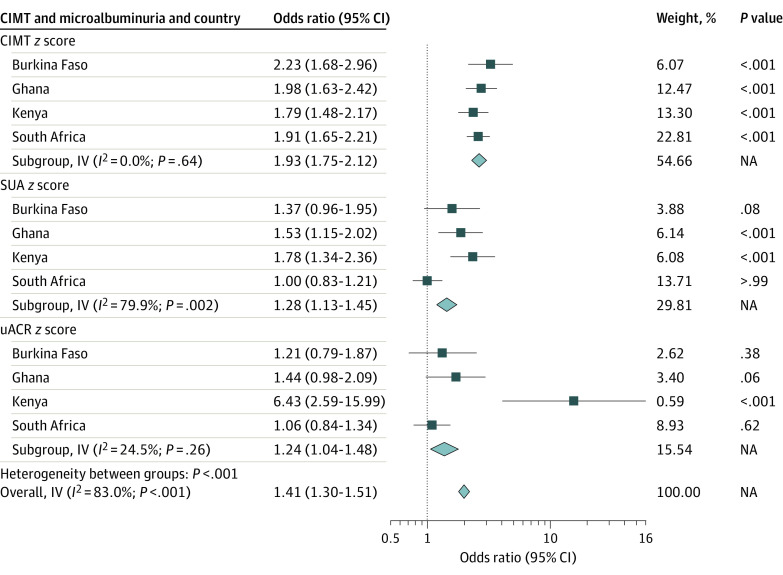
Association of Carotid Atherosclerosis and Microalbuminuria With 10-Year Atherosclerotic Cardiovascular Disease (ASCVD) Risk in the Various Countries for Women CIMT indicates carotid intima-media thickness; IV, inverse variance; NA, not applicable; SUA, spot urine albumin; uACR, urine albumin-creatinine ratio.

**Figure 3.**
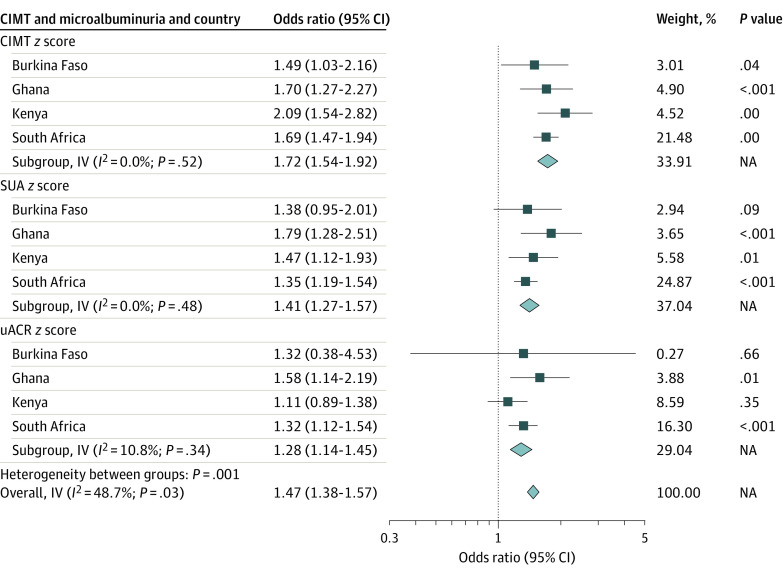
Association of Carotid Atherosclerosis and Microalbuminuria With 10-Year Atherosclerotic Cardiovascular Disease (ASCVD) Risk in the Various Countries for Men CIMT indicates carotid intima-media thickness; IV, inverse variance; NA, not applicable; SUA, spot urine albumin; uACR, urine albumin-creatinine ratio.

The fully adjusted model was thus used in meta-analyses to compare associations of carotid atherosclerosis and microalbuminuria with 10-year ASCVD risk for the various countries stratified by sex (Figure 2 and Figure 3). For instance, among women in Burkina Faso, carotid atherosclerosis was associated with a 2-fold increased risk of microalbuminuria (OR, 2.23; 95% CI, 1.68-2.96). This observed association was similar but higher than observed in Ghana (OR, 1.98; 95% CI, 1.63-2.42), South Africa (OR, 1.91; 95% CI, 1.65-2.21), and Kenya (OR, 1.79; 95% CI, 1.48-2.17). Among men, similar observations were made with Kenya (OR, 2.09; 95% CI, 1.54-2.28), with a stronger association than that observed in Ghana (OR, 1.70; 95% CI, 1.27-2.27), South Africa (OR, 1.69; 95% CI, 1.47-1.94), and Burkina Faso (OR, 1.49; 95% CI, 1.03-2.16). These analyses show that across all countries, microalbuminuria and carotid atherosclerosis were significantly associated with 10-year ASCVD risk in women and men. However, the magnitude of these associations differed between the countries and sex. In most instances, the strength of association of CIMT with ASCVD risk was higher compared with SUA and uACR.

## Discussion

The present study found that microalbuminuria was associated with CIMT independent of the traditional ASCVD risk factors. We also found that carotid atherosclerosis and microalbuminuria were associated with high 10-year ASCVD risk in middle-aged adults from sub-Saharan Africa. The results from this study offer new insights on factors that can be used to identify individuals at a high risk of ASCVD.

The findings showed differences in the prevalence of carotid atherosclerosis and microalbuminuria between countries. Participants from Kenya and South Africa reported high levels of microalbuminuria compared with those from Burkina Faso and Ghana, while the opposite was observed for common CIMT.^27^ The higher prevalence of microalbuminuria in Kenya and South Africa may be because of the higher prevalence of cardiometabolic diseases and HIV infection. The higher prevalence of alcohol use in Burkina Faso and Ghana^28^ may result in chronic liver disease and hence low circulatory serum albumin and creatinine levels. Studies involving HIV-exposed and HIV-naive individuals in rural South Africa and Hawaii reported HIV as an important predictor of microalbuminuria.^29,30^ This may be because HIV nephropathy and associated immune complex kidney disease leads to increased leakiness of nephrons to creatinine and albumin.^31,32^

The present study found that higher educational attainment was associated with lower levels of carotid atherosclerosis and microalbuminuria. Similar associations have been reported in previous studies and may reflect the association between poor education and higher levels of cardiovascular risk factors.^33,34^

We observed that SUA had a stronger association with CIMT than did uACR, as has been reported previously.^35,36,37^ The underlying mechanisms for the observed association remain unclear. However, vascular endothelial damage may cause atherosclerosis and albuminuria.^9,38^ Endothelial dysfunction increases vascular leakiness in target vessels. If these endothelial changes occur in the glomerular capillaries, the associated increased permeability in the glomerular barrier will cause increased excretion of albumin into the urine.^39^

Carotid atherosclerosis and microalbuminuria were associated with high 10-year ASCVD risk in women and men. Large epidemiological studies such as the Kuopio Ischaemic Heart Disease Risk Factor Study, Atherosclerosis Risk in Communities study, and the Cardiovascular Health Study have made similar observations with myocardial infarction and stroke.^21,40,41,42^ The Rotterdam and USE Intima-Media Thickness studies recently reported a significant association of CIMT with ASCVD risk.^43,44^ While CIMT and microalbuminuria were associated with ASCVD, microalbuminuria is more apparent in individuals with hypertension and T2D, as evident in our data. Confirming this, a review of studies in Europe, North America, and Australia observed microalbuminuria occurs in the elderly (5%), those with hypertension (11%-17%), and those with T2D (20%-30%).^45^ Among individuals with diabetes and those with advanced age, biochemical alterations to the extracellular matrix may lead to glomerulosclerosis and premature atherosclerosis, while insulin resistance leads to endothelial structural and functional damage.^46^

Our study has a number of strengths. Owing to different units of measurements, the use of *z* scores was an appropriate statistical method to enable comparisons of the magnitude of the association of carotid atherosclerosis and microalbuminuria with 10-year ASCVD risk. The updated 2018 PCE equation has an improved accuracy among all races and sex subgroups.^12^ We however acknowledge that the PCE was developed using data from African American participants and there may be underlying differences with populations in Africa owing to environmental, biological, behavioral, and clinical factors. The use of a centralized laboratory and centralized training of researchers minimized data variability. The country-level meta-analysis is a unique strength of this study, as it enabled us to compare differences in these associations across countries in different stages of epidemiological, demographic, and nutrition transition.

### Limitations

This study has limitations. The cross-sectional design prevents us from establishing causality. Social desirability and recall bias may also arise from the self-reported responses to certain questions such as previous history of CVD, smoking, and alcohol use. In addition, owing to limitations in access to health care in populations within this study it is feasible that some participants did have a history of CVD of which they were unaware. This is important to note, as the PCE was developed for use in individuals without established CVD. Additionally, the absence of clinical events data affects the observed associations, and hence, the results must be interpreted with caution.

## Conclusions

In this study, the presence of SUA may indicate subclinical carotid atherosclerosis and high 10-year ASCVD risk in middle-aged individuals from sub-Saharan Africa. These data suggest that SUA may be used to assess ASCVD risk in both sexes, and with limited regional variation in our results, SUA could be used in many countries across Africa. However, these results should be confirmed in longitudinal studies with cardiovascular event data within African populations.

## Notes

Supplement. **eFigure.** Association of Microalbuminuria (Spot Urine Albuminuria and Urine Creatinine-Albumin Ratio) With Carotid Intima-Media Thickness by the Study Countries

## References

[1] Roth GA, Johnson C, Abajobir A, . Global, regional, and national burden of cardiovascular diseases for 10 causes, 1990 to 2015. J Am Coll Cardiol. 2017;70(1):1-25. doi:10.1016/j.jacc.2017.04.052 28527533 PMC5491406

[2] GBD 2015 Risk Factors Collaborators. Global, regional, and national comparative risk assessment of 79 behavioural, environmental and occupational, and metabolic risks or clusters of risks, 1990-2015: a systematic analysis for the Global Burden of Disease Study 2015. Lancet. 2016;388(10053):1659-1724. doi:10.1016/S0140-6736(16)31679-8 27733284 PMC5388856

[3] Yuyun MF, Sliwa K, Kengne AP, Mocumbi AO, Bukhman G. Cardiovascular diseases in Sub-Saharan Africa compared to high-income countries: an epidemiological perspective. Glob Heart. 2020;15(1):15. doi:10.5334/gh.403 32489788 PMC7218780

[4] Gerstein HC, Mann JF, Yi Q, ; HOPE Study Investigators. Albuminuria and risk of cardiovascular events, death, and heart failure in diabetic and nondiabetic individuals. JAMA. 2001;286(4):421-426. doi:10.1001/jama.286.4.421 11466120

[5] Libby P, Ridker PM, Maseri A. Inflammation and atherosclerosis. Circulation. 2002;105(9):1135-1143. doi:10.1161/hc0902.104353 11877368

[6] Bots ML, Evans GW, Tegeler CH, Meijer R. Carotid intima-media thickness measurements: relations with atherosclerosis, risk of cardiovascular disease and application in randomized controlled trials. Chin Med J (Engl). 2016;129(2):215-226. doi:10.4103/0366-6999.173500 26830994 PMC4799550

[7] Bots ML, Sutton-Tyrrell K. Lessons from the past and promises for the future for carotid intima-media thickness. J Am Coll Cardiol. 2012;60(17):1599-1604. doi:10.1016/j.jacc.2011.12.061 22999720

[8] Sung JK, Kim JY, Youn YJ, . Urine albumin creatinine ratio is associated with carotid atherosclerosis in a community based cohort: atherosclerosis risk of rural area in Korean general population study. J Cardiovasc Ultrasound. 2010;18(4):134-138. doi:10.4250/jcu.2010.18.4.134 21253362 PMC3021891

[9] Stehouwer CD, Smulders YM. Microalbuminuria and risk for cardiovascular disease: analysis of potential mechanisms. J Am Soc Nephrol. 2006;17(8):2106-2111. doi:10.1681/ASN.2005121288 16825333

[10] Yusuf S, Joseph P, Rangarajan S, . Modifiable risk factors, cardiovascular disease, and mortality in 155 722 individuals from 21 high-income, middle-income, and low-income countries (PURE): a prospective cohort study. Lancet. 2020;395(10226):795-808. doi:10.1016/S0140-6736(19)32008-2 31492503 PMC8006904

[11] Ôunpuu S, Negassa A, Yusuf S. INTER-HEART: a global study of risk factors for acute myocardial infarction. Am Heart J. 2001;141(5):711-721. doi:10.1067/mhj.2001.114974 11320357

[12] Yadlowsky S, Hayward RA, Sussman JB, McClelland RL, Min YI, Basu S. Clinical implications of revised pooled cohort equations for estimating atherosclerotic cardiovascular disease risk. Ann Intern Med. 2018;169(1):20-29. doi:10.7326/M17-3011 29868850

[13] von Elm E, Altman DG, Egger M, Pocock SJ, Gøtzsche PC, Vandenbroucke JP; STROBE Initiative. The Strengthening the Reporting of Observational Studies in Epidemiology (STROBE) statement: guidelines for reporting observational studies. J Clin Epidemiol. 2008;61(4):344-349. doi:10.1016/j.jclinepi.2007.11.008 18313558

[14] Ramsay M, Crowther N, Tambo E, . H3Africa AWI-Gen Collaborative Centre: a resource to study the interplay between genomic and environmental risk factors for cardiometabolic diseases in four sub-Saharan African countries. Glob Health Epidemiol Genom. 2016;1(e20):e20. doi:10.1017/gheg.2016.17 29276616 PMC5732578

[15] Ramsay M, Sankoh O; as members of the AWI-Gen study and the H3Africa Consortium. African partnerships through the H3Africa Consortium bring a genomic dimension to longitudinal population studies on the continent. Int J Epidemiol. 2016;45(2):305-308. doi:10.1093/ije/dyv187 26659658 PMC5841636

[16] Ali SA, Soo C, Agongo G, . Genomic and environmental risk factors for cardiometabolic diseases in Africa: methods used for phase 1 of the AWI-Gen population cross-sectional study. Glob Health Action. 2018;11(sup2):1507133. doi:10.1080/16549716.2018.1507133 30259792 PMC6161608

[17] Touboul PJ, Hennerici MG, Meairs S, . Mannheim carotid intima-media thickness and plaque consensus (2004-2006-2011): an update on behalf of the advisory board of the 3rd, 4th and 5th Watching the Risk Symposia, at the 13th, 15th and 20th European Stroke Conferences, Mannheim, Germany, 2004, Brussels, Belgium, 2006, and Hamburg, Germany, 2011. Cerebrovasc Dis. 2012;34(4):290-296. doi:10.1159/000343145 23128470 PMC3760791

[18] Goff DC Jr, Lloyd-Jones DM, Bennett G, . 2013 ACC/AHA guideline on the assessment of cardiovascular risk: a report of the American College of Cardiology/American Heart Association Task Force on Practice Guidelines. J Am Coll Cardiol. 2014;63(25 Pt B)(25 Pt B):2935-2959. doi:10.1016/j.jacc.2013.11.005 24239921 PMC4700825

[19] Naqvi TZ, Lee MS. Carotid intima-media thickness and plaque in cardiovascular risk assessment. JACC Cardiovasc Imaging. 2014;7(10):1025-1038. doi:10.1016/j.jcmg.2013.11.014 25051948

[20] Bian L, Xia L, Wang Y, . Risk factors of subclinical atherosclerosis and plaque burden in high risk individuals: results from a community-based study. Front Physiol. 2018;9:739. doi:10.3389/fphys.2018.00739 29988372 PMC6023999

[21] Chambless LE, Heiss G, Folsom AR, . Association of coronary heart disease incidence with carotid arterial wall thickness and major risk factors: the Atherosclerosis Risk in Communities (ARIC) Study, 1987-1993. Am J Epidemiol. 1997;146(6):483-494. doi:10.1093/oxfordjournals.aje.a009302 9290509

[22] Price JF, Tzoulaki I, Lee AJ, Fowkes FG. Ankle brachial index and intima media thickness predict cardiovascular events similarly and increased prediction when combined. J Clin Epidemiol. 2007;60(10):1067-1075. doi:10.1016/j.jclinepi.2007.01.011 17884603

[23] Derhaschnig U, Kittler H, Woisetschläger C, Bur A, Herkner H, Hirschl MM. Microalbumin measurement alone or calculation of the albumin/creatinine ratio for the screening of hypertension patients? Nephrol Dial Transplant. 2002;17(1):81-85. doi:10.1093/ndt/17.1.81 11773468

[24] Halbesma N, Kuiken DS, Brantsma AH, . Macroalbuminuria is a better risk marker than low estimated GFR to identify individuals at risk for accelerated GFR loss in population screening. J Am Soc Nephrol. 2006;17(9):2582-2590. doi:10.1681/ASN.2005121352 16899519

[25] Kidney Disease: Improving Global Outcomes (KDIGO) CKD Work Group. KDIGO 2012 Clinical Practice Guideline for the Evaluation and Management of Chronic Kidney Disease. Kidney Int Suppl. 2013;3(1):1-150. Accessed March 14, 2022. https://kdigo.org/wp-content/uploads/2017/02/KDIGO_2012_CKD_GL.pdf

[26] Stevens PE, Levin A; Kidney Disease: Improving Global Outcomes Chronic Kidney Disease Guideline Development Work Group Members. Evaluation and management of chronic kidney disease: synopsis of the kidney disease: improving global outcomes 2012 clinical practice guideline. Ann Intern Med. 2013;158(11):825-830. doi:10.7326/0003-4819-158-11-201306040-00007 23732715

[27] George JA, Brandenburg J-T, Fabian J, ; AWI-Gen and the H3Africa Consortium. Kidney damage and associated risk factors in rural and urban sub-Saharan Africa (AWI-Gen): a cross-sectional population study. Lancet Glob Health. 2019;7(12):e1632-e1643. doi:10.1016/S2214-109X(19)30443-7 31708144 PMC7033368

[28] Boua PR, Soo CC, Debpuur C, ; as members of AWI-Gen and the H3Africa Consortium. Prevalence and socio-demographic correlates of tobacco and alcohol use in four sub-Saharan African countries: a cross-sectional study of middle-aged adults. BMC Public Health. 2021;21(1):1126. doi:10.1186/s12889-021-11084-1 34118914 PMC8196437

[29] Tongma C, Shikuma CM, Nakamoto BK, . Albuminuria as a marker of cardiovascular risk in HIV-infected individuals receiving stable antiretroviral therapy. Hawaii J Med Public Health. 2013;72(9)(suppl 4):34-38. 24052917 PMC3764546

[30] Wensink GE, Schoffelen AF, Tempelman HA, Rookmaaker MB, Hoepelman AI, Barth RE. Albuminuria Is associated with traditional cardiovascular risk factors and viral load in HIV-infected patients in rural South Africa. PLoS One. 2015;10(8):e0136529. doi:10.1371/journal.pone.0136529 26309226 PMC4550462

[31] Szczech LA, Grunfeld C, Scherzer R, . Microalbuminuria in HIV infection. AIDS. 2007;21(8):1003-1009. doi:10.1097/QAD.0b013e3280d3587f 17457094 PMC3189480

[32] Hadigan C, Edwards E, Rosenberg A, . Microalbuminuria in HIV disease. Am J Nephrol. 2013;37(5):443-451. doi:10.1159/000350384 23615312 PMC3809894

[33] Kubota Y, Heiss G, MacLehose RF, Roetker NS, Folsom AR. Association of educational attainment with lifetime risk of cardiovascular disease: the Atherosclerosis Risk in Communities Study. JAMA Intern Med. 2017;177(8):1165-1172. doi:10.1001/jamainternmed.2017.1877 28604921 PMC5710437

[34] Green JA, Cavanaugh KL. Understanding the influence of educational attainment on kidney health and opportunities for improved care. Adv Chronic Kidney Dis. 2015;22(1):24-30. doi:10.1053/j.ackd.2014.07.004 25573509

[35] Kong X, Jia X, Wei Y, . Association between microalbuminuria and subclinical atherosclerosis evaluated by carotid artery intima-media in elderly patients with normal renal function. BMC Nephrol. 2012;13:37. doi:10.1186/1471-2369-13-37 22686733 PMC3406990

[36] Jørgensen L, Jenssen T, Johnsen SH, . Albuminuria as risk factor for initiation and progression of carotid atherosclerosis in non-diabetic persons: the Tromsø Study. Eur Heart J. 2007;28(3):363-369. doi:10.1093/eurheartj/ehl394 17132646

[37] Park HE, Heo NJ, Kim M, Choi SY. Significance of microalbuminuria in relation to subclinical coronary atherosclerosis in asymptomatic nonhypertensive, nondiabetic subjects. J Korean Med Sci. 2013;28(3):409-414. doi:10.3346/jkms.2013.28.3.409 23487182 PMC3594605

[38] Stehouwer CD. Endothelial dysfunction in diabetic nephropathy: state of the art and potential significance for non-diabetic renal disease. Nephrol Dial Transplant. 2004;19(4):778-781. doi:10.1093/ndt/gfh015 15031329

[39] Deen WM. What determines glomerular capillary permeability? J Clin Invest. 2004;114(10):1412-1414. doi:10.1172/JCI23577 15545991 PMC525751

[40] Salonen R, Salonen JT. Determinants of carotid intima-media thickness: a population-based ultrasonography study in eastern Finnish men. J Intern Med. 1991;229(3):225-231. doi:10.1111/j.1365-2796.1991.tb00336.x 2007840

[41] Chambless LE, Folsom AR, Clegg LX, . Carotid wall thickness is predictive of incident clinical stroke: the Atherosclerosis Risk in Communities (ARIC) study. Am J Epidemiol. 2000;151(5):478-487. doi:10.1093/oxfordjournals.aje.a010233 10707916

[42] O’Leary DH, Polak JF, Kronmal RA, Manolio TA, Burke GL, Wolfson SK Jr; Cardiovascular Health Study Collaborative Research Group. Carotid-artery intima and media thickness as a risk factor for myocardial infarction and stroke in older adults. N Engl J Med. 1999;340(1):14-22. doi:10.1056/NEJM199901073400103 9878640

[43] Gijsberts CM, Groenewegen KA, Hoefer IE, . Race/ethnic differences in the associations of the Framingham risk factors with carotid IMT and cardiovascular events. PLoS One. 2015;10(7):e0132321. doi:10.1371/journal.pone.0132321 26134404 PMC4489855

[44] Iglesias del Sol A, Bots ML, Grobbee DE, Hofman A, Witteman JC. Carotid intima-media thickness at different sites: relation to incident myocardial infarction: the Rotterdam Study. Eur Heart J. 2002;23(12):934-940. doi:10.1053/euhj.2001.2965 12069447

[45] Pollak J, Sypniewska G. Microalbuminuria and risk of cardiovascular diseases in patients with diabetes and hypertension. Biochem Med (Zagreb). 2007;18(1):25-34.

[46] Damsgaard EM, Frøland A, Jørgensen OD, Morgensen CE. Prognostic value of urinary albumin excretion rate and other risk factors in elderly diabetic patients and non-diabetic control subjects surviving the first 5 years after assessment. Diabetologia. 1993;36(10):1030-1036. doi:10.1007/BF02374495 8243851

