# Supplementary material for: Carotid Atherosclerosis, Microalbuminuria, and Estimated 10-Year Atherosclerotic Cardiovascular Disease Risk in Sub-Saharan Africa
Source: JAMA Netw Open. 2022 Apr 26;5(4):e227559. doi: 10.1001/jamanetworkopen.2022.7559 (PMC9044117; doi:10.1001/jamanetworkopen.2022.7559)
Supplement: Supplement. — eFigure. Association of Microalbuminuria (Spot Urine Albuminuria and Urine Creatinine-Albumin Ratio) With Carotid Intima-Media Thickness by the Study Countries [file jamanetwopen-e227559-s001.pdf]

Supplemental Online Content

Nonterah EA, Boateng D, Crowther NJ, et al. Carotid atherosclerosis, microalbuminuria, and estimated 10-year atherosclerotic cardiovascular disease risk in sub-Saharan Africa. *JAMA Netw Open.* 2022;5(4):e227559. doi:10.1001/jamanetworkopen.2022.7559

eFigure. Association of Microalbuminuria (Spot Urine Albuminuria and Urine Creatinine-Albumin Ratio) With Carotid Intima-Media Thickness by the Study Countries

This supplemental material has been provided by the authors to give readers additional information about their work.

© 2022 Nonterah EA et al. *JAMA Network Open.*

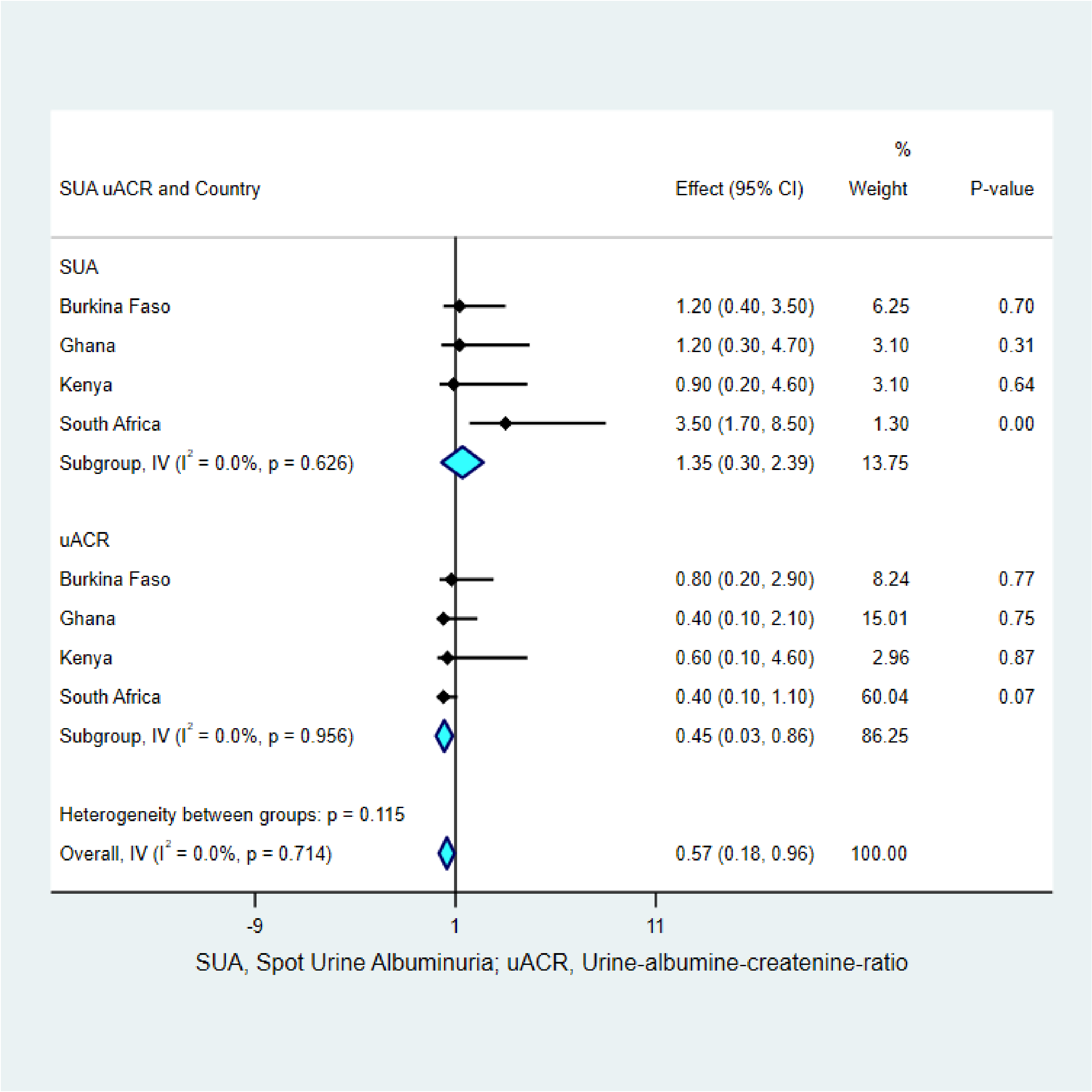

eFigure 1. Association of Microalbuminuria (Spot Urine Albuminuria and Urine Creatinine-Albumin Ratio) With Carotid Intima-Media Thickness by the Study Countries

IV, inverse variance; SUA, spot urine albuminuria; uACR, urine albumin-creatinine-ration

© 2022 Nonterah EA et al. *JAMA Network Open.*
